# MRI-based interpretable radiomics nomogram for discrimination between Brucella spondylitis and Pyogenic spondylitis

**DOI:** 10.1016/j.heliyon.2023.e23584

**Published:** 2023-12-13

**Authors:** Parhat Yasin, Yasen Yimit, Dilxat Abliz, Muradil Mardan, Tao Xu, Aierpati Yusufu, Xiaoyu Cai, Weibin Sheng, Mardan Mamat

**Affiliations:** a Department of Spine Surgery, The First Affiliated Hospital of Xinjiang Medical University, Urumqi, Xinjiang, 830054, China; b Department of Radiology, The First People’s Hospital of Kashi Prefecture, Kashi, Xinjiang, 844000, China; c Department of Orthopedic, The Eighth Affiliated Hospital of Xinjiang Medical University, Urumqi, Xinjiang, 830054, China; d Xinhua Hospital Affiliated to Shanghai Jiaotong University School of Medicine, Department of Spine Center, Shanghai, 200092, China

**Keywords:** Pyogenic spondylitis, Brucella spondylitis, Radiomics, Nomogram, SHAP

## Abstract

**Background:**

Pyogenic spondylitis (PS) and Brucella spondylitis (BS) are commonly seen spinal infectious diseases. Both types can lead to vertebral destruction, kyphosis, and long-term neurological deficits if not promptly diagnosed and treated. Therefore, accurately diagnosis is crucial for personalized therapy. Distinguishing between PS and BS in everyday clinical settings is challenging due to the similarity of their clinical symptoms and imaging features. Hence, this study aims to evaluate the effectiveness of a radiomics nomogram using magnetic resonance imaging (MRI) to accurately differentiate between the two types of spondylitis.

**Methods:**

Clinical and MRI data from 133 patients (2017–2022) with pathologically confirmed PS and BS (68 and 65 patients, respectively) were collected. We have divided patients into training and testing cohorts. In order to develop a clinical diagnostic model, logistic regression was utilized to fit a conventional clinical model (M1). Radiomics features were extracted from sagittal fat-suppressed T2-weighted imaging (FS-T2WI) sequence. The radiomics features were preprocessed, including scaling using Z-score and undergoing univariate analysis to eliminate redundant features. Furthermore, the Least Absolute Shrinkage and Selection Operator (LASSO) was employed to develop a radiomics score (M2). A composite model (M3) was created by combining M1 and M2. Subsequently, calibration and decision curves were generated to evaluate the nomogram's performance in both training and testing groups. The diagnostic performance of each model and the indication was assessed using the receiver operating curve (ROC) with its area under the curve (AUC). Finally, we used the SHapley Additive exPlanations (SHAP) model explanations technique to interpret the model result.

**Results:**

We have finally selected 9 significant features from sagittal FS-T2WI sequences. In the differential diagnosis of PS and BS, the AUC values of M1, M2, and M3 in the testing set were 0.795, 0.859, and 0.868. The composite model exhibited a high degree of concurrence with the ideal outcomes, as evidenced by the calibration curves. The nomogram's possible clinical application values were indicated by the decision curve analysis. By using SHAP values to represent prediction outcomes, our model’s prediction results are more understandable.

**Conclusions:**

The implementation of a nomogram that integrates MRI and clinical data has the potential to significantly enhance the accuracy of discriminating between PS and BS within clinical settings.

## 1 Introduction

Brucella spondylitis (BS) results from *Brucella* widespread infection in the local spine, which is the most prevalent manifestation of *Brucellar* osteomyelitis [1]. In the Mediterranean, North America, and the Middle East, brucellosis is a prevalent infectious illness. Globally, 5–6 million people globally have brucellosis and 500,000 new cases are recorded each year, according to previously released data [2,3]. The northeast and northwest regions of China, where the animal husbandry sector is well-developed, report varying rates of brucellosis, ranging from 6% to 58%. The prevalence of the condition is rising in cities and towns, though, as dairy product consumption rises [[4], [5], [6]]. Pyogenic spondylitis (PS), which accounts for 2–7% of all bone and joint infections and has an annual incidence of 0.2–2 cases/10 million individuals, is well recognized as a severe hazard to human health. Due to the increasing clinical usage of antibiotics in China, typical early cases of PS are uncommon, making early diagnosis more challenging [7]. Brucella spondylitis (BS) and PS display common non-specific clinical symptoms, including but not limited to fever, sweating, weakness, anorexia, headache, and localized backache. Then, it would bring about some difficulties in differential diagnosis at their acute or subacute stage. Delayed or missed diagnosis without appropriate treatment may come across spinal cord damage [8].

The value of MRI in diagnosing spinal infection is extensively studied [[9], [10], [11]]. Diagnostic MRI findings of spinal infection include bone marrow edema, vertebral collapse, aberrant disc signal intensity, and paraspinal or epidural extension of infection [12]. Early studies have focused on MRI predominance in differentiating BS from TS [[13], [14], [15]]. Both PS and BS can present with varying degrees of bone and disc destruction, as well as the presence of abscesses in the paraspinal or psoas regions. However, despite its sensitivity and usefulness, the diagnosis of spinal infections using MRI can be complicated due to the subjective nature of radiologists' experience. This can create challenges for junior radiologists when interpreting the imaging results. Furthermore, current MRI imaging tools face challenges in terms of the lack of specificity in differentiating between various types of infections and the limited ability to visualize small lesions. Additionally, the inability to accurately assess deep tissue involvement, such as abscesses within muscles, can hinder a comprehensive evaluation of the extent of the infection. Machine learning (ML) algorithms can overcome these limitations by learning complex patterns from large datasets objectively and mathematically.

Radiomics is a non-invasive and interdisciplinary approach that involves various qualitative techniques that can extract useful information from medical images. This method can be beneficial in both the diagnosis and prognostication of patient outcomes [16]. Radiomics is a collaborative approach that combines artificial intelligence and medical imaging to extract a high-dimensional array of quantitative variables from medical images. This innovative method can capture relevant pathophysiological information and support radiologists in various aspects of clinical practice [17,18]. SHAP (Shapley Additive exPlanations) is an extension of the Shapley value used in cooperative game theory. It is a method for determining the contribution of each feature to machine learning. The effect of adding each feature is calculated for all possible combinations by comparing the projected values with and without the inclusion of each feature and then averaging the results.

This study aims to examine the applicability of a radiomics nomogram in distinguishing between Brucella Spondylitis (BS) and Pyogenic Spondylitis (PS), particularly in their acute and subacute phases. The study seeks to integrate clinical factors and radiomics features derived from standard MRI data, allowing for an individualized differentiation of the two spondylitis manifestations.

## 2 Methods

### 2.1 Population and study design

For five years, ranging from January 2017 to December 2022, a retrospective cohort study was conducted on patients who had been diagnosed with either Brucella Spondylitis (BS) or Pyogenic Spondylitis (PS). The study included participants with an average age of 51.5 ± 12.9 years for BS and 46.3 ± 18.9 years for PS, as shown in Table 1. As part of the standard diagnostic protocol, all patients underwent various routine assessments, including blood culture, tuberculin test, T-spot TB test, standard tube agglutination titer test, and chest X-ray. The demographic and clinical features of the patients were obtained from the Electronic Medical Record System. Establishing a primary diagnosis of BS required verifying the presence of clinical manifestations, imaging features, and laboratory results.

**Table 1:** Demographic and clinical characteristics of PS and BS patients.

	All (N = 133)	BS (N = 65)	PS (N = 68)	*P*
Age (year)	49.7 ± 15.3	51.5 ± 12.9	46.3 ± 18.9	0.162
Gender (n%):				0.301
Female	56 (42.1%)	23 (35.4%)	33 (48.5%)	
Male	77 (57.9%)	42 (64.6%)	35 (51.5%)	
BMI (kg/m2)	24.1 ± 4.86	24.6 ± 5.08	23.0 ± 4.27	0.108
Pain (n%):				0.877
High	76 (57.1%)	38 (58.5%)	38 (55.9%)	
Low	57 (42.9%)	27 (41.5%)	30 (44.1%)	
Wasting (n%):				0.530
No	88 (66.3%)	45 (69.2%)	43 (60.6%)	
Yes	45 (33.7%)	20 (30.8%)	25 (39.4%)	
Affected Limb (n%):				0.695
Left	35 (26.5%)	20 (30.8%)	15 (22.1%)	
None	75 (56.1%)	34 (52.3%)	41 (60.3%)	
Right	23 (17.3%)	11 (16.9%)	12 (17.6%)	
Muscle Strength (n%):				0.193
3	56 (41.8%)	24 (36.9%)	32 (51.5%)	
4	53 (39.8%)	30 (46.2%)	23 (27.3%)	
5	24 (18.4%)	11 (16.9%)	13 (21.2%)	
WBC (×10^9^/L)	7.39 ± 2.95	6.16 ± 1.63	9.12 ± 4.10	<0.001
ESR (mm/H)	46.0 ± 19.0	43.3 ± 17.7	57.5 ± 23.9	0.004
CRP (mg/L)	36.4 ± 25.5	28.1 ± 27.9	58.6 ± 42.9	0.001
ALB (g/L)	37.3 ± 4.97	37.5 ± 4.26	36.9 ± 6.19	0.640
AST (U/L)	28.5 ± 15.3	27.1 ± 14.3	31.4 ± 17.0	0.211
ALT (U/L)	32.4 ± 20.3	31.2 ± 20.6	34.7 ± 19.8	0.409
GGT (U/L)	48.3 ± 25.9	48.5 ± 25.4	47.7 ± 27.3	0.880
ALP (U/L)	100 ± 25.6	101 ± 27.0	98.0 ± 22.7	0.521

PS: Pyogenic spondylitis, BS: Brucella spondylitis, BMI: Body mass index (BMI, kg/m^2^), WBC: preoperative white blood cell (WBC, ×10^9/^L), ESR: preoperative erythrocyte sedimentation rate (ESR, mm/h), CRP: preoperative C-reactive protein (CRP, mg/L), ALB: pre-operative operative albumin (ALB, g/L), AST: preoperative aspartate aminotransferase (AST, U/L), ALT: preoperative alanine aminotransferase (ALT, U/L), GGT: preoperative gamma-glutamyl transferase (GGT, U/L), Alkaline phosphatase (ALP, U/L).

The study's inclusion criteria involved the following aspects: 1) present relevant clinical symptoms for at least six months; 2) positive agglutination test with a titer of 1/160 or higher, and/or blood culture, and/or the identification of Brucella spp. from the relevant tissue samples, or histopathological examination [14,15]; 3) presence at least one morphologic and signal abnormality on MRI; 4) complete medical records.

The exclusion criteria for this investigation included the following: 1) the presence of relevant clinical symptoms for less than six months without any prior treatment before undergoing MRI; 2) incomplete MRI acquisition, such as missing T2-weighted imaging or fat-suppressed T2WI; 3) a lack of clinical or histopathological data of the patients under investigation; 4) poor image quality resulting from motion or susceptibility artifacts; 5) spondylitis caused by tuberculosis, malignant tumors, lumbar disc herniation, non-bacterial infectious etiology (such as fungal infections), noninfectious (inflammatory or neoplastic) lesions, unidentified infectious pathogen, or incomplete information regarding the infectious agent involved in the condition.

**Fig. 1:**
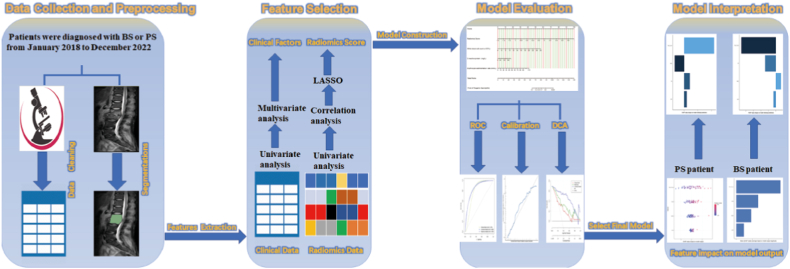
**Flowchart of this research.** The flowchart demonstrates the sequential steps involved in the research, including data collection and preprocessing, feature extraction, feature selection, model development, model evaluation, and model interpretation.

To develop the clinical-feature-alone model, the radiomics signature model, and the combined clinical-radiomic model. All enrolled patients were segregated into two separate datasets known as the training dataset (composed of 93 patients) and the testing dataset (comprising 40 patients), distributed in a ratio of 7:3. Predictive nomograms were developed for each of these models. Fig. 1 provides an overview of the entire analysis process. This study obtained approval from the institutional review board, and written informed consent was deemed unnecessary as the study was retrospective in nature.

### 2.2 MR protocols

The imaging devices utilized in this study were 1.5 T MR scanners manufactured by Siemens Healthcare located in Erlangen, Germany. The study employed a range of imaging sequences, including T1-weighted images (T1WI) in sagittal sequences, T2-weighted images (T2WI) in axial and sagittal sequences, and fat-suppressed T2-weighted images (FS-T2WI) in sagittal sequences. The primary image parameters for each sequence were T1WI (repetition time [TE] = 600 ms; echo time [TE] = 9.5 ms; field of view [FOV] = 320 mm; 256 × 256 matrix), T2WI (repetition time [TE] = 3000 ms; echo time [TE] = 88 ms; field of view [FOV] = 320 mm; 256 × 256 matrix), and FS-T2WI (repetition time [TE] = 3600 ms; echo time [TE] = 83 ms; field of view [FOV] = 320 mm; 256 × 256 matrix), all incorporating a slice thickness of 4 mm and a slice spacing of 1 mm. For feature extraction, sagittal FS-T2WI was selected as the imaging sequence of choice. The digital imaging and communication in medicine (DICOM) formatted images were acquired through the picture archiving and communication system (PACS).

### 2.3 Radiologic evaluation and segmentation

Two experienced radiologists, specializing in spine MRI with 5 and 3 years of relevant experience, thoroughly assessed the MR pictures. The radiologists utilized the open resource software 3D-Slicer (version 4.8.1) to segment the lesions on each layer and identify regions of interest, while remaining blinded to the clinical data (ROIs) **(see** Supplementary file 1**)**. Independently, they examined the images twice, with a 2-week gap between examinations, without prior knowledge of the histological findings. Intraclass correlation coefficients (ICCs) were employed to calculate both inter- and intra-observer agreements. For future research, radiomic characteristics exhibiting exceptional stability, along with inter- and intra-observer ICC values greater than 0.75, were selected [19].

### 2.4 Radiomics feature extraction

Using the Pyradiomics package, version 2.2.0, which can be downloaded at https://pyradiomics.readthedocs.io, radiomic characteristics were extracted from MRI images. The original feature classes were derived directly from the unmodified MR images, capturing inherent characteristics without any additional alterations. On the other hand, the filter classes included features obtained after applying various filters such as wavelet, square, square root, logarithm, etc. to the original MR images. This classification scheme ensured a comprehensive assessment of the radiomic characteristics by investigating both the unfiltered original features and the impact of different filters on feature extraction.

### 2.5 Features selection and radiomics signature building

While feature redundancy may reduce classification accuracy, the availability of high-dimensional features raises computing costs. Prior to feature selection, normalization was carried out to ensure consistency in the value scales of the radiomic features. Z-score normalization was chosen to normalize the radiomics features to a 0–1 scale, preventing any variations in the features' variances， which was utilized in our research [20]. After conducting a robustness test on the gathered features, those with an intraclass correlation coefficient (ICC) agreement score greater than 0.75 were judged stable and included in further study [21]. The *Mann-Whitney U* test was performed on the stable features to determine significant variables between the BS and PS groups. Following that, Pearson correlation matrices were used to evaluate the correlation between the characteristics. Characteristics were deemed redundant if their correlation coefficients were higher than 0.8. As high-dimensional features could lead to overfitting in the learning algorithms, and some features may be irrelevant to the task of spondylitis classification. In order to decrease the number of features and determine the most relevant characteristics, we employed the LASSO logistic regression method, which utilizes a least absolute shrinkage and selection operator. The ratio of training cohort to testing cohort was 7 : 3. Tuning parameter λ was determined using 10-fold cross-validation on the training dataset (93 cases), minimizing the binomial deviances [22]. This approach aims to determine the optimal number of features that exert a more significant influence on the diagnosis of PS. To that end, the LASSO logistic regression model was employed, and its performance was refined through a penalty parameter tuning process that involved 10-fold cross-validation using minimum criteria. For each patient, a radiomics score (rad-score, Rad Score), which reflects the relevance of each feature according to their respective LASSO coefficients, was computed. The formula for the Rad-score is as follows: Rad-score (Rad Score) = β0 + β1Feature1 + β2Feature2 + … + βn*Feature n. In this formula, β0 is the intercept or constant term, βn represents the regression coefficients for each selected feature, and Feature1 … Feature n are the radiomic features selected for a given patient. By substituting the corresponding values for each feature into the formula, the linear combination of the selected features is calculated, resulting in the Rad-score for that patient. The LASSO method was implemented using the “glmnet” package within the R software platform [23].

### 2.6 Development and verification of personalized clinical-radiomics nomograms

By univariate logistic regression analysis, we first assessed the prognostic value of clinical factors. Multivariate logistic regression analysis was then used to integrate parameters with *P* < 0.05 in order to create the nomogram model. To illustrate the effect of the radiomics score and other clinical factors on the individual risk of PS, we created a nomogram based on chosen variables. Calibration curves were employed in both the training and test datasets to evaluate the diagnostic performance of the combined nomogram. Additionally, the receiver operating characteristic (ROC) curve, produced using bootstrap resampling (with 5000 iterations), and the area under the curve (AUC) value were used to assess discrimination performance [24]. The Delong nonparametric approach was then applied to compare the AUC estimates obtained from the prediction models [25]. In order to evaluate the calibration of the radiomics model, calibration curves were created using bootstrapping with 500 resamples. Furthermore, we also plot the predicted probability-density plot to investigate the theoretical and actual classification of the final model. Finally, we constructed three models: clinical features model 1 (M1), radiomics signature (Rad Score) Model 2 (M2), and combining M1 and M2 as combined model 3 (M3).

### 2.7 The clinical relevance and interpretation of the combined model

We employed a decision curve analysis (DCA) to compute the net benefits for various threshold probabilities, evaluating the therapeutic utility of the nomogram. Furthermore, we employed the SHAP (Shapley Additive exPlanations) framework in the “fastshap” package [26] to analyze the effects of internal features on the model output. The SHAP value quantifies the contribution of each feature (or predictor) to the output of a machine learning model, offering insights into the influence of each feature on the prediction for a specific instance [27]. Based on cooperative game theory, SHAP values calculate the average marginal contribution of each feature across all possible feature combinations.

### 2.8 Statistical analysis

We started our study by using Q-Q plots to apply Normal Distribution tests to all continuous variables. Non-normal variables were presented as a median and interquartile range, while those with a normal distribution were displayed as mean ± SD. We used statistical tests based on the characteristics of the variables to see whether there was any discernible difference between the two groups. For categorical variables, we specifically utilized the Chi-square test. The *Mann-Whitney U* test was used for variables with non-normal distribution, whereas Student's *t*-test was used for variables with normal distribution. The threshold for statistical significance was defined as a two-tailed P value of 0.05 or lower. All statistical analyses were conducted using R software (version 4.2.0; R Foundation for Statistical Computing; http://www.r-project.org).

## 3 Results

### 3.1 Clinical features analysis

Patients' demographic and medical features were shown in tabular form (Table 1). A total of 38 BS patients were excluded from the study due to various reasons, including clinical symptoms <6 months (n = 9), incomplete MRI acquisition (n = 15), lack of clinical/histopathological data (n = 10), and poor image quality (n = 4). Additionally, 30 PS patients were excluded for reasons such as incomplete MRI acquisition (n = 4), lack of clinical/histopathological data (n = 13), poor image quality (n = 3), and other spinal conditions (n = 10). Subsequently, we analyzed the data of 65 BS patients and 68 PS patients, comparing their demographic and clinical characteristics. The findings indicated a higher likelihood of BS development among elderly patients; however, the difference was not statistically significant. There were no significant differences observed between PS and BS patients regarding wasting symptoms, muscle strength, or hepatic function indices (*P* = 0.695, *P* = 0.193, and *P* = 0.193, respectively).

### 3.2 Feature reduction and radiomics signature building

The data from the training group were analyzed using multivariate logistic regression analysis. Parameters with statistically significant differences were utilized to develop the clinical features model 1 (M1), as presented in Table 2. It was determined that WBC and ESR were independent predictors of PS. (*P* < 0.01) Additionally, CRP was included in the model to investigate its clinical implications.

**Table 2:** Univariate and multivariate analysis of clinical predictions factors for PS patients.

	Univariate analysis			Multivariate analysis		
Characteristic	OR	95% CI	*P*	OR	95% CI	*P*
Age	0.97	0.93, 1.00	0.080			
Gender						
Female	1.00	–				
Male	0.71	0.25, 1.99	0.517			
BMI	0.93	0.82, 1.04	0.205			
Pain (n%)						
High	1.00	–				
Low	1.18	0.42, 3.29	0.756			
Wasting (n%)						
No	1.00	–				
Yes	1.38	0.45, 4.08	0.563			
Affected Limb (n%)						
Left	1.00	–				
None	0.97	0.28, 3.67	0.962			
Right	0.90	0.17, 4.61	0.899			
Muscle Strength (n%)						
3	1.00	–				
4	0.31	0.08, 1.00	0.059			
5	0.88	0.22, 3.42	0.853			
WBC (×10^9^/L)	1.59	1.20, 2.26	0.004	1.42	1.01, 2.15	0.065
ESR (mm/H)	1.03	1.01, 1.07	0.013	1.03	1.00, 1.07	0.085
CRP (mg/L)	1.03	1.01, 1.05	0.002	1.01	0.98, 1.03	0.5
ALB (g/L)	0.95	0.85, 1.06	0.353			
AST (U/L)	1.01	0.97, 1.05	0.556			
ALT (U/L)	1.01	0.98, 1.03	0.695			
GGT (U/L)	1.00	0.98, 1.02	0.939			
ALP (U/L)	0.99	0.96, 1.01	0.205			

PS: Pyogenic spondylitis, OR: Odds Ratio, CI: Confidence Interval, BMI: Body mass index (BMI, kg/m^2^), WBC: preoperative white blood cell (WBC, ×10^9/^L), ESR: preoperative erythrocyte sedimentation rate (ESR, mm/h), CRP: preoperative C-reactive protein (CRP, mg/L), ALB: pre-operative operative albumin (ALB, g/L), AST: preoperative aspartate aminotransferase (AST, U/L), ALT: preoperative alanine aminotransferase (ALT, U/L), GGT: preoperative gamma-glutamyl transferase (GGT, U/L), Alkaline phosphatase (ALP, U/L).

**Fig. 2:**
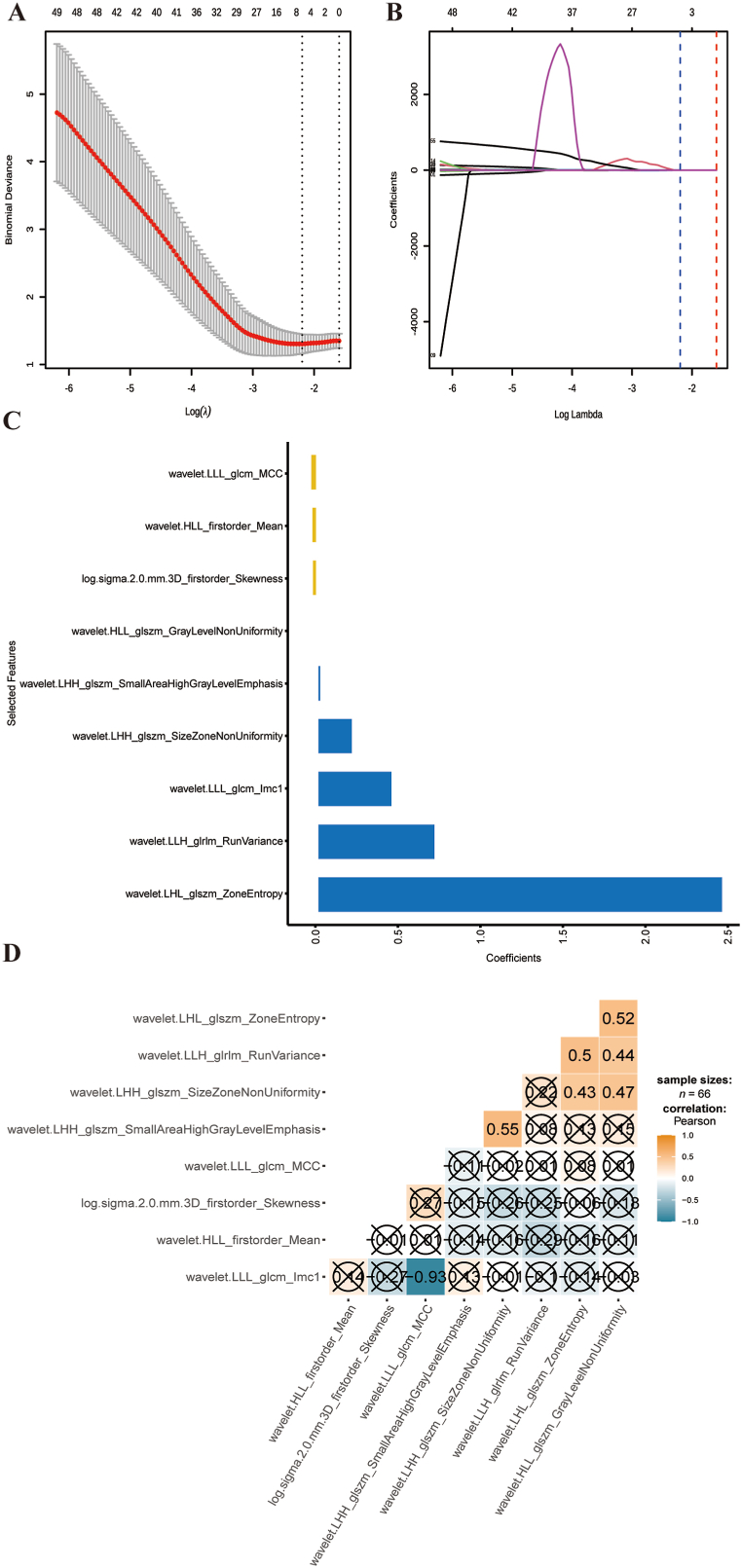
**Feature selection and establishment of radiomics score (Rad Score).** (A) The process of choosing the best parameter (lambda) using ten-fold cross-validation, with the minimal criteria and the 1-SE of the minimum criteria indicated by vertical lines. (B) The Least Absolute Shrinkage and Selection Operator (LASSO) coefficient profiles reveal the nine features with nonzero coefficients. (C) Corresponding coefficients for the selected feature.

**Fig. 3:**
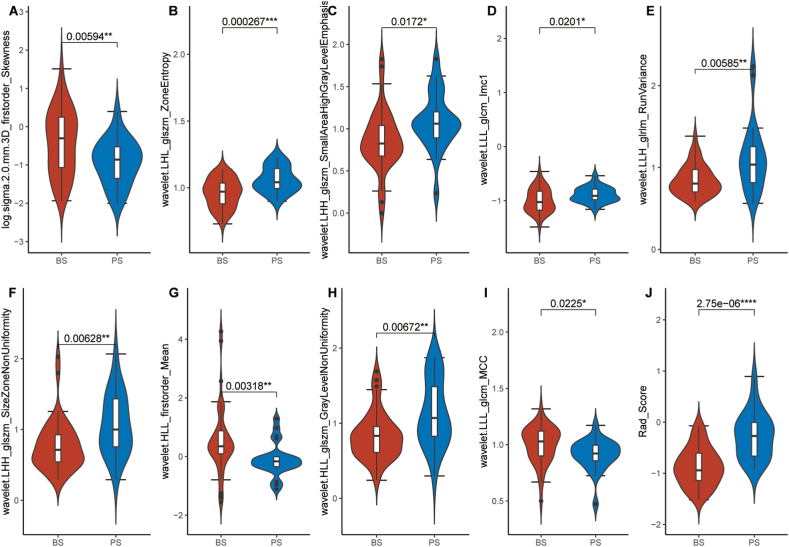
**Illustrates a comparative evaluation of the selected radiomics features and radiomics signature (Rad Score).** Violin plots are used to depict the distribution of radiomics features (A–I) and radiomics scores (J) characterized by the types of spondylitis: Brucella spondylitis (BS) and Pyogenic spondylitis (PS). A two-sample *t*-test was used to determine whether there was a significant difference between the means of the two groups.

**Fig. 4:**
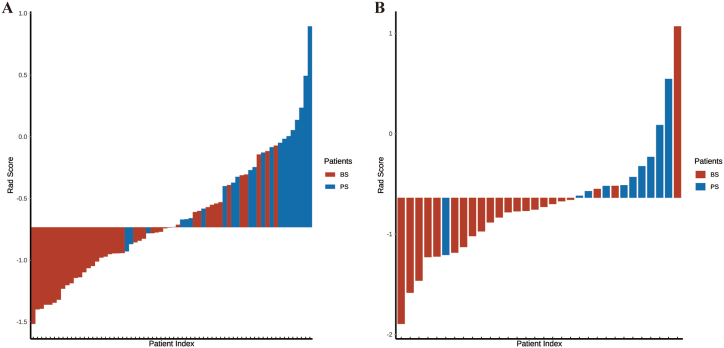
**The radiomics signature value of enrolled cases.** Radiomics signature distribution for each patient in training set (A) and testing set (B).

A total of 1585 features were extracted from areas of interest in this research (ROIs) and underwent univariate analysis, resulting in 1390 remaining features. To mitigate collinearity, highly correlated variables (r ≥ 0.85) were eliminated, as illustrated in Fig. 2D. Consequently, 301 features remained after the removal of correlated features. Following that, binomial deviances and coefficients with different tuning factors (*λ*) were determined (Fig. 2A and B). Nine radiomics features with non-zero coefficients were chosen from 301 features obtained from the T2WI-FS method based on the optimum *λ* value of 0.111 (Fig. 2C). Between the BS and PS groups, these nine characteristics showed a significant variation (*P* < 0.05), as illustrated in Fig. 3A–I. The radiomics signature (Rad Score) for PS patients was found to be statistically higher than for BS patients (*P* < 0.05). This signature was derived from the nine selected features and their corresponding coefficients (Fig. 3H). The distributions of the radiomics signature for each subject in both the training and testing datasets are shown in Fig. 4A and B, respectively. Radiomics signature (Rad Score) was used to establish model 2 (M2).

### 3.3 Assessment of individual risk nomogram

**Fig. 5:**
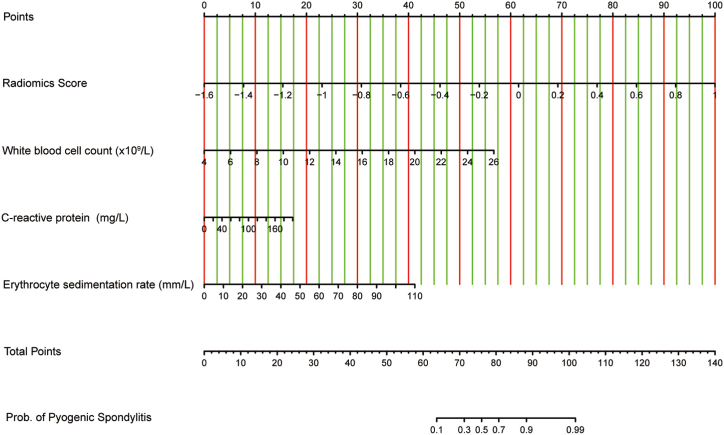
**Visualization of the combined model with the Rad-score and clinical characteristics.** The radiomics nomogram, which includes the radiomics signature (Rad Score) along with white blood cell count (WBC), erythrocyte sedimentation rate (ESR), and C-reactive protein (CRP), was created using logistic regression analysis in the training cohort.

**Fig. 6:**
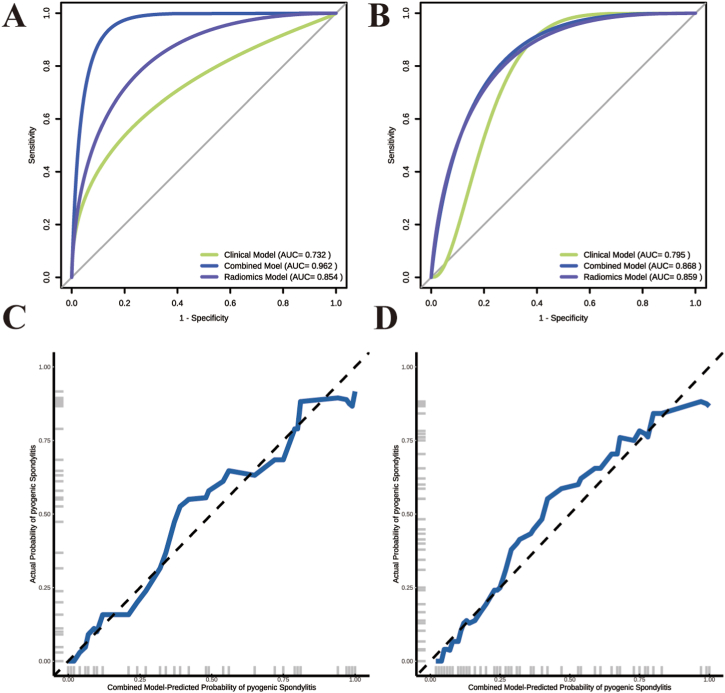
**Portrays the receiver operating characteristic (ROC) curves and calibration curves.** (A) ROC curves for the training dataset. (B) ROC curves for the testing dataset. (C) Calibration curves for the training dataset. (D) Calibration curves for the testing dataset.

A combined nomogram (M3) was created using logistic regression analysis by combining the radiomics signature (Rad Score) and identifying clinical traits, including WBC, ESR, and CRP, as independent risk variables (Fig. 5). Subsequently, ROC curve was utilized to evaluate the performance of the clinical model (M1), radiomics signature (M2), and combination model (M3), as depicted in Fig. 6A and B. The results demonstrated that M3 exhibited the best performance, achieving the highest AUC values of 0.962 and 0.868 in the training and testing groups, respectively. In the training and testing datasets, the M2 attained AUC values of 0.857 and 0.859, respectively. M1 exhibited inferior performance compared to the M2 and M3 models, with AUC values of 0.732 and 0.795 in the training and testing datasets, respectively. Moreover, as shown in Fig. 6C and D, calibration curves showed high agreement between the estimated and real risk of PS.

### 3.4 Discriminatory analysis of each model

**Fig. 7:**
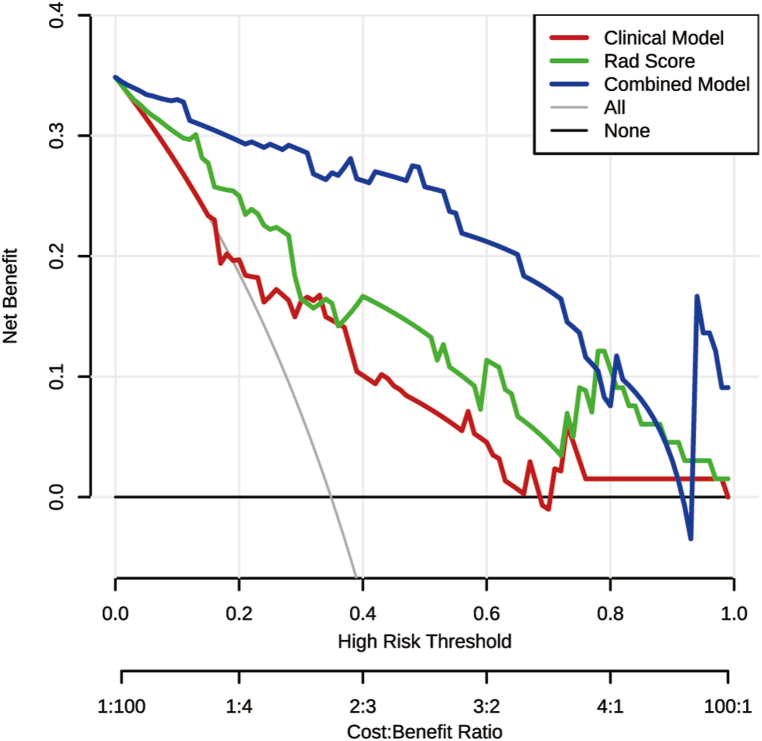
**Decision curve analysis (DCA) curves for the clinical model, radiomics model and combined model.** The decision curve analysis (DCA) was performed to compare the clinical features-based model (red line), the radiomics features-based model (green line), and the joint model (blue line) that included both clinical and radiomics features.

Based on the analysis presented in Table 3, when compared to M1 and M2 working independently, the combined model (M3) demonstrated the highest diagnostic efficiency. The Delong test provided additional evidence of substantial differences between M1, M2 and M3 (*P* = 0.001 and *P* = 0.007, respectively). In Fig. 7, the DCA curves of the combined model demonstrate that the M3 model outperformed the treat-all-patients and treat-none strategies in recognizing PS. Throughout the interval where a patient or doctor's threshold probability was below 0.795 or above 0.004, the net advantage of M3 was greater or on par with the other two models.

**Table 3:** Differentiation performance of different models.

Models	Sen.	Spe.	Acc.	Npv.	Ppv.	Rec.	P*
Clinical Model (M1)	0.435	0.884	0.727	0.745	0.667	0.435	
Radiomics Model (M2)	0.652	0.86	0.788	0.822	0.714	0.652	
Combined Model (M3)	0.826	0.953	0.909	0.911	0.905	0.826	
M1 vs M2							0.194
M1 vs M3							0.001
M2 vs M3							0.007

Sen., sensitivity; Spe., specificity; Acc., accuracy; NPV., negative predictive value; P.P.V., positive predictive value; Rec., recall. *P: models’ comparison were finished via DeLong test.

**Fig. 8:**
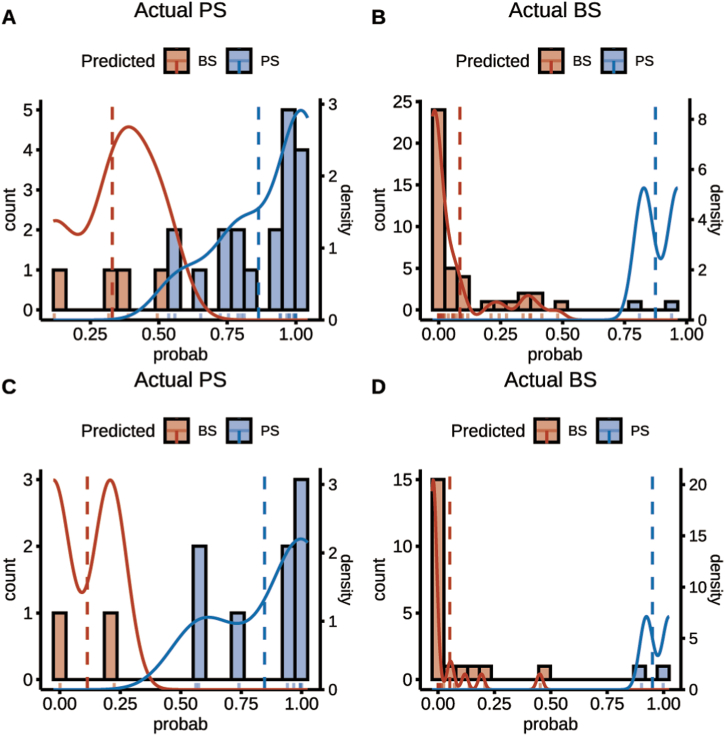
**Individual probability density distribution.** (A) Probability density distribution curve illustrating the actual observed probabilities of prolonged PS cases in the training dataset. (B) Probability density distribution curve displaying the actual observed probabilities of BS cases in the training dataset. (C) Probability density distribution curve showcasing the actual observed probabilities of prolonged PS cases in the testing dataset. (D) Probability density distribution curve depicting the actual observed probabilities of BS cases in the testing dataset.

**Fig. 9:**
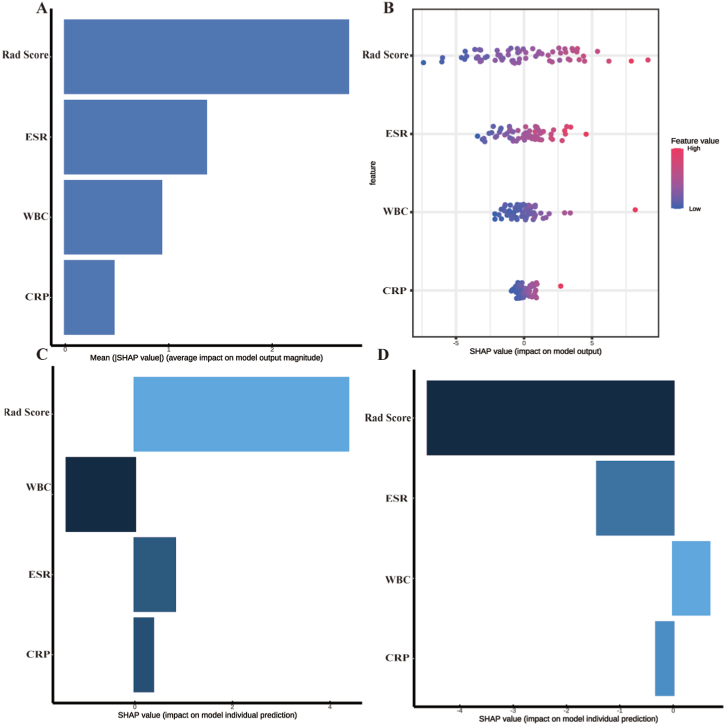
**Interpretation of combined model.** (A) Relative feature significance based on SHapley Additive exPlanations (SHAP) values. (B) Summary plots of SHAP values for the radiomic-clinical model. (C) Illustrates the relative SHAP value in identifying a patient with PS (Pyogenic spondylitis). (D) Illustrates the relative SHAP value in identifying a patient with BS (Brucella Spondylitis).

Fig. 8 presents the probability-density plot, which demonstrates approximate consistency in the distribution of the probability of each disease in both the training and testing sets. Additionally, predicted and actual labels were observed to exhibit better consistency. SHAP values were calculated for variables included in the combined model, and Fig. 9 presents the variance importance, summary, decision, and single care prediction. A positive SHAP value signified an increased risk of PS for each prediction, and vice versa for negative values. The variables at the top of the figure display higher predictive power and are regarded as more significant contributors to the model than those located at the bottom. The variance importance plot observed in Fig. 9A (A feature is more significant if it has a higher average SHAP value) indicates that the radiomics signature (Rad Score) is the most significant risk factor, as it has the highest SHAP values. Furthermore, Fig. 9B (A positive SHAP value indicates a higher risk of PS, while the opposite is true for BS) demonstrates that a higher Rad-score led to an increased probability of PS diagnosis. Fig. 9C and D emphasized the decisive role of the Rad Score in single prediction.

## 4 Discussion

This study aimed to differentiate BS and PS through clinical and radiomics approaches. Our results showed that the integrated clinical and radiomics model has the best efficiency in distinguishing these spinal infections. Based on published studies, the frequency of spinal infections in Western nations is progressively on the rise and has now escalated to 6.5 occurrences per 100,000 people annually. These infections comprise 5% of all bone infections and are the dominant type of hematogenous osteomyelitis in elderly individuals [28,29]. In conjunction with the widespread misuse of intravenous drugs and the aging of Western society, there is a growing roster of patients who are immunocompromised, diabetic, reliant on dialysis, or possess permanent vascular access. These high-risk populations are more likely to experience repeated bacteremia episodes and subsequent hematogenous spine seeding. Moreover, diagnostic tools, especially MRI, are more accessible [30,31]. Infectious spondylitis, according to pathogenic infection can be divided into-parasitic spondylitis, pyogenic spondylitis, and granulomatous spondylitis. Parasitic spondylitis is rare (spondylitis caused by metastases of parasites to the spine, like cystic and alveolar echinococcosis), and clinically spondylitis mainly refers to septic spondylitis, brucellosis spondylitis, and tuberculosis spondylitis [3,32].

To the best of our knowledge, this is the first study to use MRI-based radiomics and clinical information to differentiate between BS and PS using an interpretable machine-learning algorithm (logistic regression). Our research incorporates nine radiomics features and laboratory diagnostics, including the measurement of three distinct inflammatory markers: erythrocyte sedimentation rate (ESR), C-reactive protein (CRP), and white blood cell (WBC) count. Our combined model (clinical indicators + radiomics features) displayed better discrimination ability with AUC values: 0.962, and 0.868 in training datasets and testing datasets respectively. Based on the general interpretation of the result obtained from our model using the SHAP method, we found Rad Score was the top important factor.

The erythrocyte sedimentation rate (ESR) has been identified as a laboratory biomarker with high sensitivity for infection, showing a positive result in over 90% of patients with spinal infections. Our investigation has shown average ESR levels of 43.3 ± 17.7 and 57.5 ± 23.9 mm per hour in BS and PS, respectively. Pyogenic spondylitis produces an acute infection in the spine and tends to grow quickly. A large and immediate inflammatory response brought on by the bacteria's fast reproduction causes an increase in ESR. Contrarily, Brucella spondylitis often develops slowly, frequently requiring months to manifest. Due to this chronicity, the inflammation is less severe and the ESR is thus lower [33,34]. In addition to differentiating PS and BS, Chiang HY et al. have found ESR is closely related to treatment duration and recurrence of PS [35].

The acute-phase protein known as C-reactive protein is produced by hepatocytes (CRP). While minute concentrations of this protein are present in healthy individuals’ serum, levels increase within 6 h of the onset of bacterial infection. In terms of diagnostic specificity, CRP is increased in 90% or more individuals with spinal infections, compared to ESR [12]. Although both CRP and ESR levels are typically elevated following spinal surgery or infection, CRP returns to baseline more quickly after timely treatment or surgical intervention, in comparison to ESR. In our study, mean CRP levels among patients with BS and PS were 28.1 ± 27.9 and 58.6 ± 42.9 g per liter, respectively. Davis WT et al. have discovered that high CRP cut-off values above normal levels are high for pyogenic spondylitis, which is in line with our findings [36].

Elevations in CRP and/or ESR are not conclusive evidence of infection, but they can be helpful in screening and monitoring spinal infections. Our study revealed a substantial alteration in white blood cell (WBC) count between patients with Brucella spondylodiscitis (BS) ((6.16 ± 1.63) × 10^9^/L) and pyogenic spondylitis (PS) ((9.12 ± 4.10) × 10^9^/L). Although WBC count may not be highly specific in diagnosing spinal infections, it should still be included in the diagnostic workup for fever and suspected infections as it can provide broader insight into a patient's response to treatment [37]. Lee et al. discovered that CRP and WBCs were significantly decreased after 4 weeks of antibiotic treatment in pyogenic spondylitis patients, which can be used as a biomarker that early evaluate treatment effect [38]. Similarly, these two biomarkers also can be used to differentiate PS and BS, which was discovered in our study. Our research findings are consistent with previous studies that recommend considering elevated white blood cell count (WBC), C-reactive protein (CRP), and erythrocyte sedimentation rate (ESR) as important indicators when confirming the diagnosis of spinal infectious diseases [39,40].

In addition to investigating the role of these biomarkers in differentiating PS and BS, we have evaluated features selected from T2WI-FS sequences. Out of 1585 features extracted from sagittal T2WI-FS sequences, nine were selected for the construction of a radiomics signature. Notably, first-order skewness, wavelet HLL first-order mean, wavelet LLL glcm MCC BS were higher than in PS; whereas wavelet LHL glszm zone entropy, wavelet LHH glszm small area high gray level emphasis, wavelet LLH glrlm run variance, wavelet LHH glszm size zone non-uniformity, wavelet H LL glszm gray level non-uniformity were significantly higher in PS. Consequently, the constructed radiomics signature showed higher values in PS compared to BS. Entropy, a statistical indicator of tissue heterogeneity, quantifies the textural irregularity in the histogram, and has been extensively studied in radiomics research. Previous studies have demonstrated that entropy can distinguish malignant and benign breast tumors, indicating its potential crucial role in differentiating PS from BS [41].

Recent studies have investigated the performance of CT or MRI-based radiomic features in spinal infectious sickness (i.e., BS, TS, and PS) [13,42]. To our knowledge, there was a lack of literature on discerning MRI features that can accurately distinguish between Brucella spondylitis (BS) and pyogenic spondylitis (PS) during their acute and subacute stages. Furthermore, previous radiomics studies have predominantly focused on model construction and performance, with limited attention given to model interpretations. In our efforts to address this, we utilized Shapley additive explanations (SHAP), a framework that can estimate and compute the contribution of each feature (input) to the result [43]. SHAP values capture how individual features impact predictions by quantifying their influence on the difference between the model's predicted output and the average prediction. This facilitates an intuitive understanding of feature impact, whether visualizing the importance of features in a single prediction or aggregating across multiple predictions to rank global feature importance. Moreover, the model-agnostic nature of the SHAP approach allows it to be applied to any black-box machine learning model, including deep learning, decision trees, and random forests. This unified framework enhances transparency and interpretability, thereby facilitating fair model comparison and evaluation.

In the field of medical image analysis, researchers have explored various avenues, including image classification, segmentation, and lesion identification. Image classification algorithms, such as convolutional neural networks (CNNs), have demonstrated remarkable success in categorizing medical images into distinct classes, aiding in rapid and accurate disease identification [44]. Additionally, segmentation algorithms, like U-Net, Mask Region-based Convolutional Neural Network (R-CNN) and You Only Look Once (YOLO) have proven effective in outlining and delineating regions of interest within medical images, facilitating precise localization of abnormalities [45]. Building on our research findings, there are promising avenues for future research. One such direction involves integrating auto-segmentation techniques with radiomics-based diagnosis. By automating the segmentation process using advanced algorithms like U-Net, we can improve the efficiency of radiomics analysis. This allows for the extraction of a wide range of quantitative features from medical images, leading to more accurate and personalized diagnoses. Furthermore, the automation of lesion identification holds tremendous potential. Developing algorithms like Mark R-CNN, and You Only Look Once (YOLO) capable of automatically identifying and characterizing lesions in spine images can significantly expedite the diagnostic process and improve overall patient outcomes. By leveraging auto-segmentation and automated lesion identification techniques, we believe we can enhance the accuracy, efficiency, and speed of medical diagnoses, benefiting both patients and healthcare professionals.

There are some limitations in our study, First, even though we have independently verified the model, however, this was not multicenter research, the robustness and reproducibility of the radiomics nomogram require independent testing using additional datasets from multi-medical facilities. Second, this is a retrospective study, even though we have strict inclusion and exclusion criteria, selection bias is inevitable. Finally, even though we have collected materials in 5 years, due to the rarity of PS, this study included a relatively small sample size, future large-scale multi-center studies are needed to further investigate the performance of this model.

## 5 Conclusion

In conclusion, our research suggests that MRI-based radiomics nomograms have potential in the differential diagnosis of BS and PS. The combination of radiomics analysis with clinical factors could provide a more accurate and efficient diagnostic tool for clinicians.

## Ethical approval

This study was approved by the Ethics Committee of the Xinjiang Medical University Affiliated First Hospital (K202309-15) and waived the requirement of written informed consent for participation.

## Data availability statement

The data associated with our study was not deposited into a publicly available repository. Data will be made available on request. Data will be made available on request.

## Funding

This work was supported by the 10.13039/501100015310Xinjiang Uygur Autonomous Region Natural Science Foundation Youth Science Foundation Project (Grant Number 2022D01C745).

## CRediT authorship contribution statement

**Parhat Yasin:** Writing - original draft, Visualization, Software, Methodology, Formal analysis. **Yasen Yimit:** Writing - review & editing, Validation, Resources, Conceptualization. **Dilxat Abliz:** Writing - review & editing, Visualization, Resources. **Muradil Mardan:** Visualization, Investigation, Formal analysis. **Tao Xu:** Validation, Resources. **Aierpati Yusufu:** Visualization, Validation. **Xiaoyu Cai:** Validation, Supervision. **Weibin Sheng:** Visualization, Validation. **Mardan Mamat:** Writing - review & editing, Validation, Supervision, Resources, Investigation.

## Declaration of competing interest

The authors declare that they have no known competing financial interests or personal relationships that could have appeared to influence the work reported in this paper.
