# Supplementary figures and images for: MRI-based interpretable radiomics nomogram for discrimination between Brucella spondylitis and Pyogenic spondylitis
Source: Heliyon. 2023 Dec 13;10(1):e23584. doi: 10.1016/j.heliyon.2023.e23584 (PMC10761805; doi:10.1016/j.heliyon.2023.e23584)

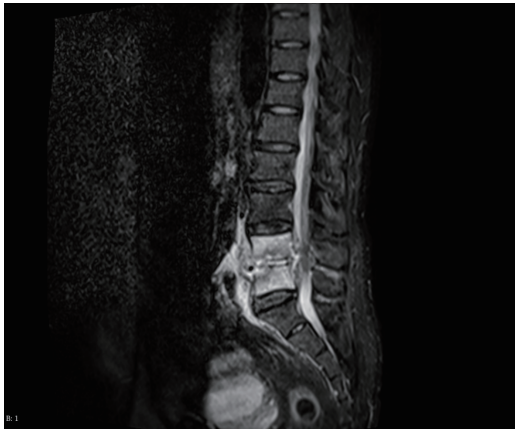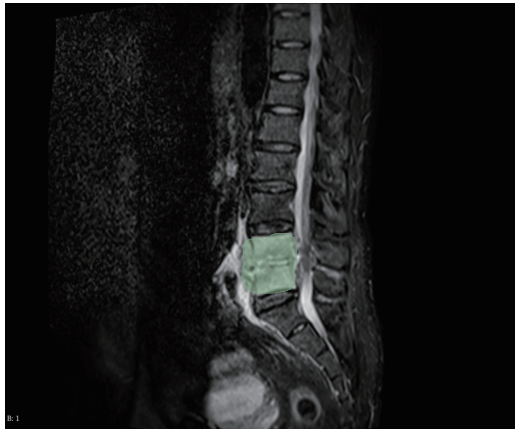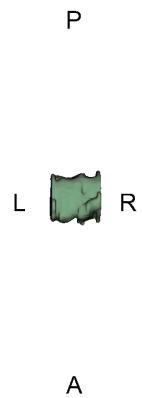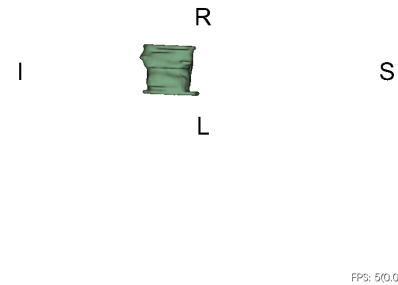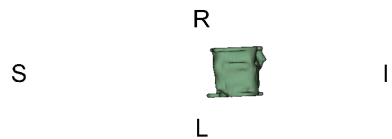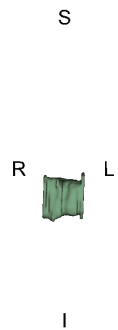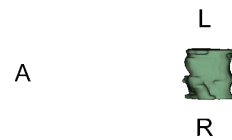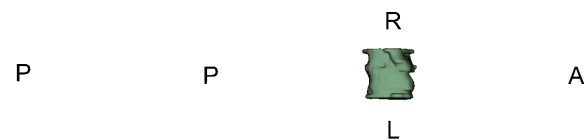

Supplement: Multimedia component 1 [file mmc1.pdf]
